# Fidelity of DNA ligase I is sensitive to physiological Mg^2+^ level

**DOI:** 10.1016/j.jbc.2026.111407

**Published:** 2026-03-25

**Authors:** David H. Beier, Eunhye Lee, Ihn Sik Seong, Vanessa C. Wheeler, Patrick J. O’Brien

**Affiliations:** 1 Department of Biological Chemistry, University of Michigan, Ann Arbor, Michigan, USA; 2 Molecular Neurogenetics Unit, Center for Genomic Medicine, Massachusetts General Hospital, Boston, Massachusetts, USA; 3 Department of Neurology, Massachusetts General Hospital and Harvard Medical School, Boston, Massachusetts, USA; 4 Medical and Population Genetics Program, the Broad Institute of M.I.T. and Harvard, Cambridge, Massachusetts, USA

**Keywords:** DNA ligation, DNA ligase fidelity, Huntington’s disease, LIG1 syndrome, ligase fidelity, magnesium

## Abstract

The pool of free intracellular Mg^2+^ varies among tissues with the highest concentration measured in muscle tissue and the lowest measured in immune cells and in the brain. Here we investigate the impact of free Mg^2+^ on the fidelity of human DNA ligase I (LIG1). LIG1 is the major DNA ligase and is required to complete DNA replication, recombination and repair pathways. Biallelic hypomorphic variants of LIG1 cause immunodeficiency-96. We employed steady-state kinetics to compare fidelity of LIG1 towards a damaged nucleobase at the 3′- hydroxyl side of a nicked DNA substrate. The fidelity for discrimination between a damaged and undamaged nick increases by 21-fold when the free Mg^2+^ concentration is decreased from 1.0 to 0.2 mM. This has important implications for neurodegenerative and immune diseases, because the brain and the immune system are reported to have free Mg^2+^ concentration in the range from 0.2 to 0.4 mM. We examined a recently characterized minor variant of LIG1, K845N, which has a protective effect in Huntington’s disease, and found that the fidelity of K845N LIG1 is also enhanced as free Mg^2+^ decreases. This increase in fidelity is mainly due to the increased release of the AMP-DNA intermediate from a pro-mutagenic DNA substrate. A model is proposed whereby the fidelity of DNA transactions is sensitive to the availability of the Mg^2+^ cofactor for DNA ligation and therefore ligation fidelity may vary between tissues.

Human DNA Ligase I (LIG1) seals nicks during DNA replication and repair. Inherited deficiency in LIG1 causes LIG1 syndrome (immunodeficiency-96; MIM #619774), characterized by immunodeficiency and increased genome instability (1, 2, 3). A recent genome-wide association study identified a minor LIG1 variant (K845N; dbSNP rs145821638; GRCh38 - Chr19:48117686-C-A) to be correlated with a ∼7.7-years delay in symptomatic onset of Huntington’s disease (HD) (MIM #143100) (4). This implied beneficial effect for the K845N variant is supported by a mouse HD model, cell culture and biochemical characterization (5). The K845N variant of LIG1 exhibits higher fidelity than the WT LIG1, preferentially ligating a canonical (Watson-Crick paired nick) relative to a nick containing a mismatch which may contribute to its protective role (5).

DNA ligases have a core 3-domain architecture (6, 7) composed of an adenylylation domain (AdD), an OB-fold domain (OBD) and a DNA binding domain (DBD). These three domains completely encircle the DNA substrate ((8); Fig. S1). DNA ligases must dynamically transition between open and closed conformations to complete their catalytic cycle. The conformational dynamics of the OBD relative to the AdD have been best documented for the minimal ATP-dependent DNA ligase from Chlorella virus PBCV-1 (ChVLig), but have been observed in many DNA ligases (9). Crystal structures of ChVLig show that the AMP-DNA intermediate can exist in multiple conformations (10, 11), and solution NMR showed rapid dynamics of the OBD in the absence of DNA that are compatible with the engagement of DNA in the clamped structure (9). For human LIG1, molecular dynamics have similarly observed dynamics of the OBD (12). The interface of the OBD and AdD is highly conserved in LIG1 in animals, including K845 (5). The substitution of lysine to asparagine (K845N) is expected to break contacts with the carbonyl groups of V750 and L753. This weakened interface between the oligonucleotide binding (OBD) and the AdD is likely to cause increased enzyme flexibility and a decreased stability of the closed clamp conformation of LIG1 (Fig. S1).

Mg^2+^ ions are essential cofactors for the catalytic activity of mammalian DNA ligases. The mechanism of ATP-dependent DNA ligases is highly conserved across all domains of life (6, 13, 14). As shown in Figure 1, 2 Mg^2+^ ions are required for the reaction with ATP, with one bound to LIG1 prior to ATP binding, and another coordinated with ATP, helping stabilize negative charges and positioning the α-phosphate for nucleophilic attack by the catalytic lysine (15, 16). During the subsequent AMP transfer step, the affinity for Mg^2+^ is enhanced and limiting Mg^2+^ results in a significant drop in the rate of AMP transfer from LIG1 to the 5′-phosphate of the nicked DNA substrate (16). In the final nick sealing step, the 3′-hydroxyl group attacks the 5′-phosphate of the AMP-DNA to form the phosphodiester bond. When Mg^2+^ is limiting, all enzymatic steps are slowed, increasing the likelihood that LIG1 dissociates before AMP transfer (unproductive binding), or after AMP transfer and before nick sealing (abortive ligation). The result of abortive ligation is the formation of a non-productive AMP-DNA product that requires alternative repair pathways to be resolved, impacting both ligation efficiency and genome stability (Fig. 1; (16)).

**Figure 1:**
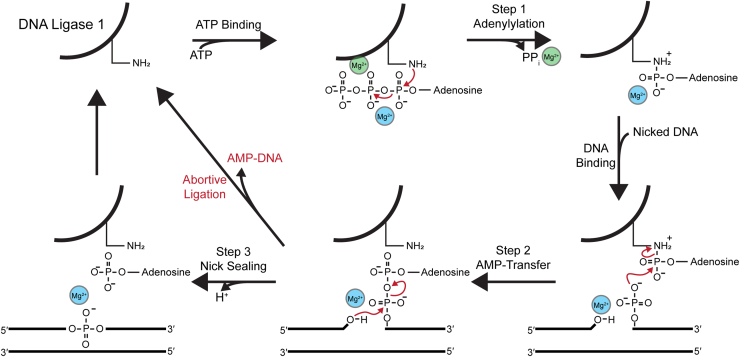
**Minimal three-step chemical mechanism for ligation by LIG1.** In the initial adenylylation step the catalytic lysine of LIG1 (depicted as -NH_2_) attacks the α-phosphate of ATP coordinated with Mg^2+^, generating a lysine–AMP intermediate and releasing pyrophosphate. Next, LIG1 binds to the nicked DNA and the anionic oxygen at the 5′-phosphate of the nick attacks the α-phosphate of lysine-AMP, resulting in an AMP-DNA intermediate. In the final enzyme-catalyzed step, the 3′-hydroxyl group upstream of the nick attacks the 5′-phosphate of the AMP-DNA, forming a phosphodiester bond to seal the nick. When Mg^2+^ is limiting, all enzymatic steps are slowed. This delay increases the chance that LIG1 dissociates from its nicked DNA substrate early, resulting in unproductive binding prior to transfer of AMP from LIG1 to the nicked substrate or abortive ligation where AMP-DNA dissociates prior to nick sealing. LIG1, DNA ligase I.

LIG1, uniquely among the three human DNA ligases, employs a Mg^2+^ ion distal to the active site to enhance fidelity with respect to mismatches on the 3′ hydroxyl (-OH) side of the nick. The binding site at the junction between the DBD and the AdD has been referred to as the HiFi site, which has much higher Mg^2+^ affinity than the catalytic Mg^2+^ binding site (15). Mutation of the conserved carboxylate ligands prevents Mg^2+^ binding and results in a higher activity and lower fidelity enzyme (15). The HiFi Mg^2+^ ion makes a direct interaction with the phosphodiester backbone at the −3 position from the nick and it appears to serve as a lynchpin in arranging the interface between the DNA, the DBD and the AdD. Thus, LIG1 uses Mg^2+^ ions for both catalysis and to provide a platform for high fidelity substrate recognition.

Magnesium frequently serves as a cofactor for DNA replication and repair enzymes. DNA polymerases and nucleases also require one or more Mg^2+^ ions to neutralize charge and position catalytic residues for nucleophilic attack (16, 17, 18, 19). For example, in Pol β, multiple Mg^2+^ ions are required for nucleotide-binding, catalysis and a conformational change to a closed state (17, 20, 21). While other divalent ions can be used, substitution of Mg^2+^ lowers fidelity (22). Studies measuring how Mg^2+^ concentration impacts fidelity of DNA repair enzymes are limited, however it has been reported that the fidelity of HIV-1 reverse transcriptase is enhanced as the free Mg^2+^ level is decreased (23, 24, 25). The physiological concentration of Mg^2+^ varies between soft tissues, with the highest concentration of free Mg^2+^ in muscle and the lowest in immune cells and the brain (26, 27, 28). Given the strong disease delaying effect of K845N LIG1 found in HD patients (4), it is important to study this enzyme variant at Mg^2+^ concentrations relevant to the brain.

Here, we investigated the impact of free Mg^2+^ concentration on the biochemical activity of WT LIG1 and the K845N LIG1 variant. Our results indicate the K845N substitution leads to a mild reduction in Mg^2+^ affinity relative to WT. Additionally, when compared to WT LIG1 under low Mg^2+^ conditions, abortive ligation increases while the rate of sealed product formation decreases. Using steady-state kinetic analysis, we compared the ligation efficiency of a canonical Watson-Crick base paired nick (C•G) with that of a pro-mutagenic 8oxoG•A lesion at the 3′-OH position. At 0.2 mM Mg^2+^ both the maximal turnover rate (k_cat_) and catalytic efficiency (k_cat_/K_M_) decrease compared to conditions at 1.0 mM Mg^2+^. However, the discrimination against ligating oxidatively damaged nicks is enhanced 13-fold for K845N and 21-fold for WT LIG1 relative to 1.0 mM free Mg^2+^ (5), suggesting that the fidelity enhancement granted by the K845N substitution is largely independent of the fidelity enhancement observed at lower concentrations of Mg^2+^. We established that this enhanced fidelity is primarily attributed to a heightened rate of release of the AMP-DNA intermediate when engaged with the pro-mutagenic substrate. These data indicate that the availability of the Mg^2+^ cofactor directly modulates the efficiency and fidelity of DNA ligation by LIG1. These findings have important implications for neurodegenerative and immune diseases; given that the brain and immune system are reported to have lower free Mg^2+^ concentrations, and LIG1 variants associated with disease typically affect neurological or immune function.

## Results

### Mg^2+^ dependence of K845N LIG1

While physiological Mg^2+^ concentrations in many tissues are estimated to be ∼1.0 mM, levels of Mg^2+^ in the brain are reported to be up to 5-fold lower (0.2–0.4 mM) (26, 27, 28). Therefore, it is important to evaluate the Mg^2+^ sensitivity of the K845N LIG1 variant in the context of physiological conditions that might be most relevant in HD pathogenesis. We predict that the K845N LIG1 substitution breaks an interdomain contact between the OBD and the AdD and destabilizes the closed complex of LIG1 encircling the DNA substrate (Fig. S1). This structural change could alter the affinity for Mg^2+^ and therefore we characterized the dependence of steady-state ligation on the concentration of Mg^2+^ ions. These assays were performed with truncated LIG1 (Δ232) that lacks the unstructured N-terminus but retains the same maximal rate of ligation (8, 16).

**Figure 2:**
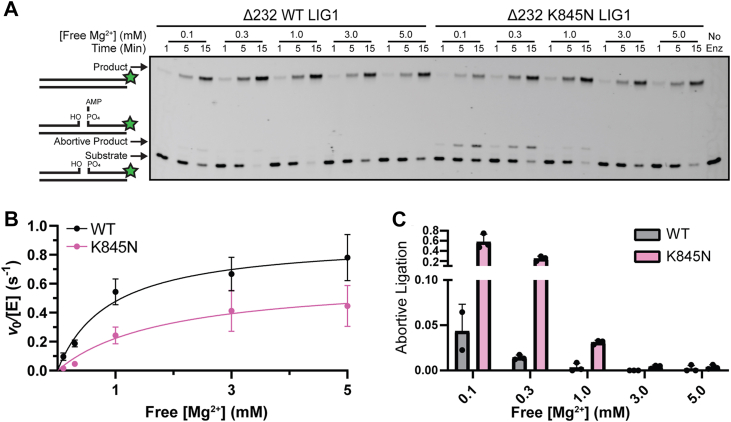
**K845N exhibits elevated abortive ligation as compared to WT LIG1.***A*, representative gel showing the difference in activity and abortive ligation for WT or K845N LIG1 with C•G nicked DNA. Reactions were performed at 37 °C with 0.2 mM ATP, variable MgCl_2_, 50 mM MOPS pH 7.5 and an ionic strength of 150 mM. *B*, steady-state dependence on Mg^2+^ concentration was fit by a rectangular hyperbola to determine the best fit values of k_cat_ and K_Mg_ values for WT or K845N LIG1. *C*, the fraction abortive ligation was calculated at each free Mg^2+^ concentration. Data reported are the average and standard deviation of at least 3 replicates and are summarized in Table S1. LIG1, DNA ligase I.

Ligation reactions were performed at 37 °C using varying concentrations of Mg^2+^ under standard buffer conditions. Steady-state assays were performed using an excess of 28mer nicked DNA substrate (1000 nM) and limiting enzyme (1 nM for Δ232 WT, 2 nM for Δ232 K845N, hereafter referred to as either WT or K845N LIG1). Representative Mg^2+^ dependent ligase assays are shown in Figure 2*A*, comparing WT and K845N LIG1 across a gradient of Mg^2+^ concentration. Initial rates of ligation for WT and K845N LIG1 were measured for a saturating concentration of nicked DNA substrate (1000 nM) at each concentration of Mg^2+^. Under all cases the reactions were linear, and triplicates were in excellent agreement (Fig. S2). Accounting for the 2-fold difference in enzyme concentration, K845N exhibited reduced ligase activity under all conditions tested. Strikingly, K845N also showed a significant amount of abortive ligation (release of AMP-DNA) at lower concentrations of Mg^2+^ (0.1–0.3 mM), which is suppressed at higher concentrations (Fig. 2*A*).

These data are plotted in Figure 2*B* and fit by a hyperbolic dependence to yield the K_1/2_ for Mg^2+^ activation (K_Mg_; Equation 4). Although LIG1 binds to multiple Mg^2+^ ions (15), this dependence fits the predicted behavior for a single essential Mg^2+^ cofactor. Given that Mg^2+^ binds weakly, we expect that it is in rapid equilibrium and K_Mg_ reflects the dissociation constant for the weakest binding of the catalytic Mg^2+^ ions. For WT LIG1, the K_Mg_ value is 1.1 ± 0.4 mM, while the K845N substitution results in a decreased affinity for Mg^2+^ at 1.8 ± 0.3 mM (Fig. 2*B*). The maximum catalytic rate (k_cat_) is 0.95 ± 0.19 s^-1^ for WT and 0.64 ± 0.23 s^-1^ for K845N, demonstrating that while K845N has a mild defect in Mg^2+^ binding, the rates of catalysis are similar under saturating Mg^2+^ conditions.

We also calculated the fraction of abortive ligation for WT and K845N LIG1 when free Mg^2+^ was varied from 0.1 to 5 mM (Fig. 2*C*). This analysis shows that the deficiency of the K845N variant is attributed to abortive ligation which reaches a level of 60% for K845N at 0.1 mM free Mg^2+^. To further support these conclusions, we repeated the Mg^2+^ dependence in a different buffer system using acetate in place of chloride. Under these conditions the K_Mg_ values for WT and K845N LIG1 were 0.85 ± 0.25 mM, and 1.8 ± 0.2 mM, respectively (Fig. S3). This is within experimental error of the values measured in the chloride-based buffer. The maximal rate of reaction is slightly lower in the acetate buffer, and this is accompanied by a modest reduction in the fraction of abortive ligation for both WT and K845N LIG1, suggesting that the anion has a minimal effect on LIG1 activity (Fig. S3). Thus, the activity of WT LIG1 is dependent upon Mg^2+^ concentration in the physiologically relevant range from 0.2 to 1.0 mM and K845N shows a decreased affinity for Mg^2+^ and elevated abortive ligation at the lower end of this range.

### Nicked DNA substrate dependence of WT and K845N LIG1

Previous studies have used 1 mM free Mg^2+^ (*e.g.*, (5, 29, 30)), which is appropriate for some cellular contexts, or super-physiological concentrations ranging from 5 to 20 mM which can mask biochemical defects (*e.g.*, (2, 12, 31, 32)). To better reflect the lower physiological concentration of Mg^2+^ in the brain, we performed a full characterization of the K845N LIG1 variant at 0.2 mM Mg^2+^ (26, 27). Previous data indicate that k_cat_ values decrease as the concentration of Mg^2+^ is reduced (15, 16), however the impact of Mg^2+^ on catalytic efficiency has not been examined. Therefore, we determined the nicked DNA substrate dependence for the reactions catalyzed by WT and K845N LIG1.

Ligation reactions were performed at 0.2 mM free Mg^2+^ at 37 °C under standard buffer conditions to determine the dependence on DNA concentration. Both a canonical C•G nicked DNA substrate and nicked substrate containing an oxidative lesion (8oxoG•A) at the 3′-OH position of the nick were used to assess the kinetic parameters of WT and K845N LIG1. The 8oxoG•A lesion is notable as it is not only the most common form of oxidative damage in DNA but is also a context that is known to be challenging for LIG1 to discriminate against (33, 34, 35). Additionally, this lesion models the oxidative stress observed in late-stage HD (36, 37, 38).

**Figure 3:**
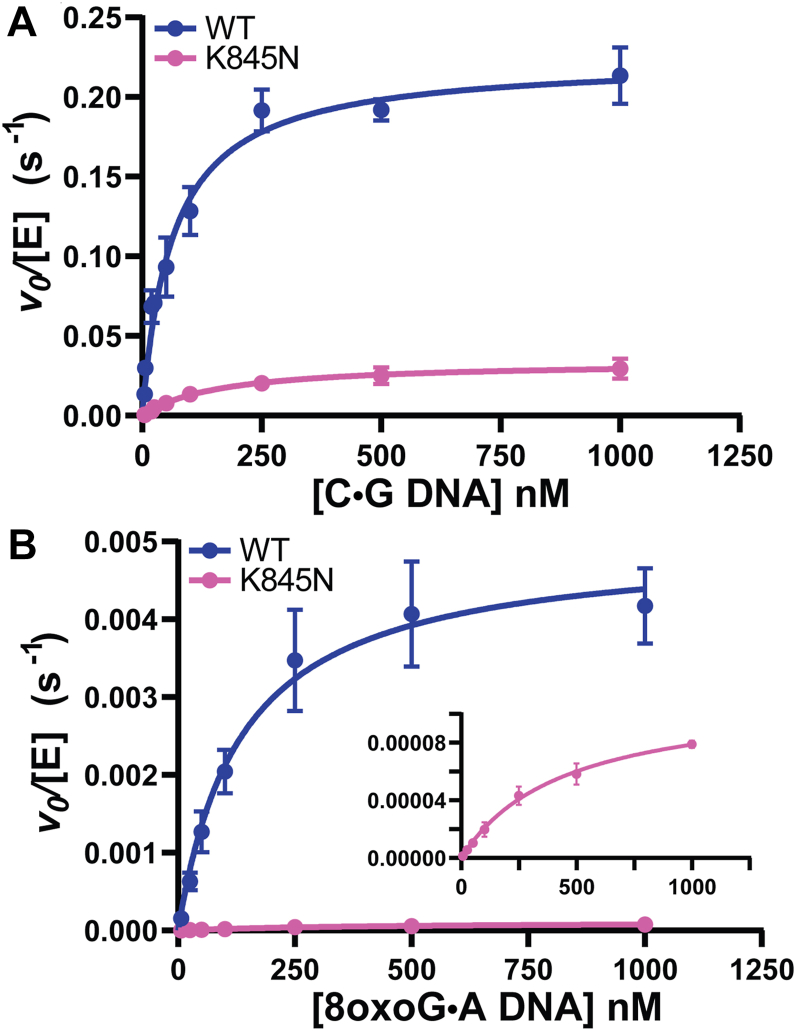
**Steady-state DNA dependence of LIG1 variants at 0.2 mM free Mg^2+^.** The C•G (*A*) or 8oxoG•A containing (*B*) nicked DNA dependence was determined with WT or K845N LIG1 in the presence of 0.2 mM ATP and 0.2 mM free Mg^2+^ (0.4 mM total Mg^2+^). Data were fit by the Michaelis-Menten equation to determine the k_cat_ and K_M_ kinetic parameters. Data reported are the average and standard deviation of at least 3 replicates and are summarized in Table S2.

**Figure 4:**
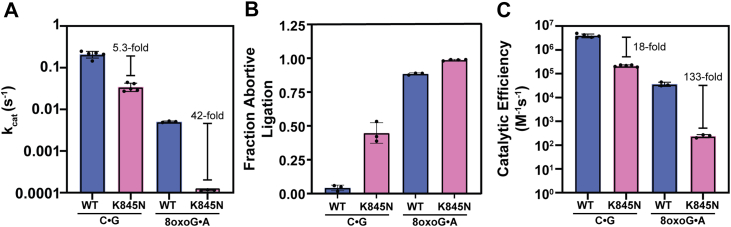
**Impact of free Mg^2+^ concentration on kinetic parameters and abortive ligation.***A*, k_cat_ values for nick sealing were determined by Michaelis-Menten kinetics at saturating nicked DNA substrate concentrations for WT and K845N LIG1 at 0.2 mM free Mg^2+^. *B*, fraction abortive ligation was calculated by Equation 2 and represents what fraction AMP-DNA intermediate LIG1 fails to seal. *C*, Michaelis-Menten fits (Fig. 3) were used to determine the catalytic efficiency of LIG1 variants sealing C•G and 8oxoG•A nicked DNA. Data reported are the average and standard deviation of at least 3 replicates and are summarized in Table S2. LIG1, DNA ligase I.

Michaelis-Menten plots for WT and K845N LIG1 acting on the canonical C•G substrate are shown in Figure 3*A*. Fitting these data with Equation 3 yields the maximal turnover number (k_cat_), the Michaelis-constant (K_M_) and the catalytic efficiency (k_cat_/K_M_). These values are reported in Table S2 and compared in Figure 4. When acting on the canonical C•G nick it is apparent that the K845N variant is significantly impaired compared to WT LIG1 at low Mg^2+^ concentration (0.2 mM) in contrast to the mild defect observed at 1.0 mM Mg^2+^ ((5); Fig. 4 and S4). The k_cat_ value for K845N LIG1 is 5.3-fold lower than WT LIG1 (Fig. 4*A*). This reduction in k_cat_ is accompanied by a 3.1-fold increase in K_M_ for K845N LIG1 (Table S2). The decrease in catalytic efficiency (k_cat_/K_M_) is 18-fold for K845N compared to WT LIG1 (Table S2 and Fig. 4*C*). This impairment in k_cat_ and k_cat_/K_M_ for the K845N substitution is greater than previously observed for measurements at 1.0 mM Mg^2+^ (5) and is likely exacerbated by the mild defect in Mg^2+^ affinity for K845N LIG1. This finding implies that the interdomain contact involving K845 is required for optimal activity at low Mg^2+^.

Michaelis-Menten plots for 8oxoG•A show a large reduction in ligation by K845N LIG1 (Fig. 3*B*). Despite this reduced activity, the initial rates of ligation are again well defined (Fig. S5) and substrate dependence fits well to the Michaelis-Menten equation (see inset of Fig. 3*B*). The resulting steady-state kinetic parameters are summarized in Table S2 and plotted for comparison in Figure 4. When acting on the 8oxoG•A substrate, K845N LIG1 has a nick sealing k_cat_ value 42-fold lower than that of WT LIG1 (Fig. 4*A*). This reduction in k_cat_ is accompanied by a 3.0-fold increase in K_M_ for K845N LIG1, reflecting a 133-fold decrease in k_cat_/K_M_ for K845N compared to WT LIG1 (Table S2; Fig. 4*C*). Decreased activity on this DNA substrate is expected to be biologically beneficial as the 8oxoG•A lesion is pro-mutagenic (*i.e.,* will result in a mutation if left unrepaired) and thus impaired ligation by the K845N LIG1 variant can serve a protective role by preventing mutagenesis.

A significant factor contributing to the reduced activity of K845N LIG1 is increased abortive ligation at physiologically relevant concentrations of Mg^2+^ (Fig. 2*C*). When acting on the canonical C•G substrate at 0.2 mM free Mg^2+^, abortive ligation remains minimal for WT LIG1 (0.04 ± 0.02) but is significantly increased by the K845N substitution (0.45 ± 0.08) (Fig. 4*B*). However, when challenged by the 8oxoG•A substrate at 0.2 mM free Mg^2+^, both LIG1 variants exhibit elevated levels of abortive ligation, with WT LIG1 failing to seal 88 ± 1% of nicks, and the K845N variant aborting the nick sealing reaction nearly every time (99 ± 0.4%) (Fig. 4*B*).

**Figure 5:**
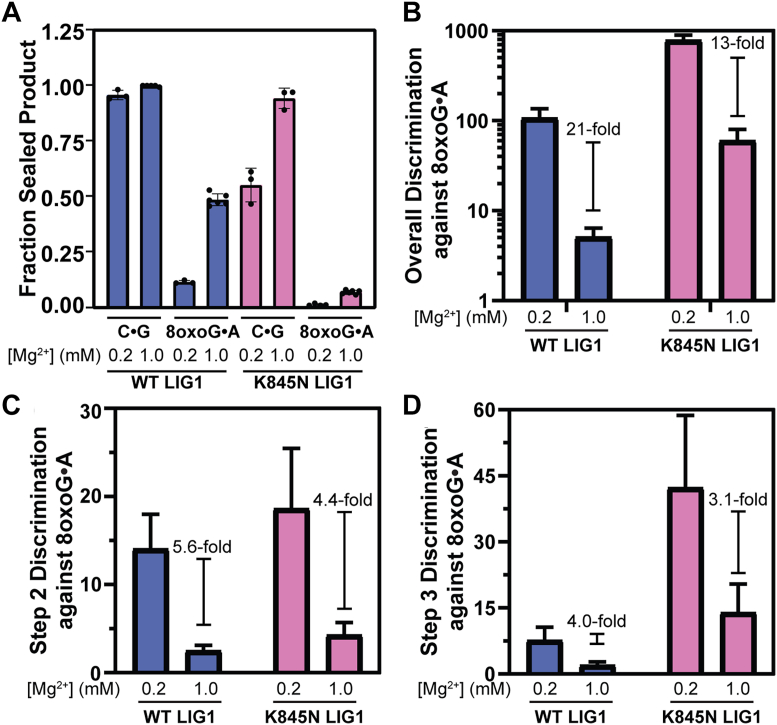
**The fidelity of LIG1 increases as free Mg^2+^ concentration decreases.***A*, fraction sealed product was calculated as (1 - fraction abortive ligation) and represents how much AMP-DNA intermediate is successfully ligated at a given concentration of Mg^2+^. *B*, catalytic efficiency (Fig. 4*C*) was used to quantitatively determine the overall discrimination against 8oxoG•A containing nicked DNA. *C*, discrimination at the AMP transfer step (step 2) was calculated using Equations (5), (6), (7), (8). *D*, discrimination at the nick sealing step (step 3) was determined using Equation 7. Data reported in *panel A* are the average and standard deviation of at least 3 replicates. Calculations reported in *panels B-D* are plotted with error bars reflecting the propagated error (see Methods; Tables S2 and S3). LIG1, DNA ligase I.

In contrast to observations made at 0.2 mM free Mg^2+^, both WT and K845N LIG1 exhibit very efficient nick sealing of the canonical C•G DNA substrate at 1.0 mM Mg^2+^ ((5); Fig. 5*A*; Table S3). When Mg^2+^ levels are reduced to 0.2 mM, the K845N variant diverges from WT LIG1, with WT maintaining high levels of nick sealing (96 ± 2%), and K845N LIG1 successfully ligating only 55 ± 8% of nicks (Fig. 5*A*). Thus, WT LIG1 is tuned to efficiently ligate canonical substrates even at the lowest concentration of Mg^2+^ found in the body, while K845N LIG1 is compromised under conditions of limited Mg^2+^ availability.

The defect in nick sealing is amplified for the 8oxoG•A substrate. At 1.0 mM Mg^2+^, WT LIG1 still maintains a moderate level of nick sealing (49%), yet K845N LIG1 exhibits a 7-fold drop in nick sealing to 7% ((5); Fig. 5*A*; Table S3). When Mg^2+^ levels are reduced from 1.0 to 0.2 mM, abortive ligation became even more pronounced, with WT LIG1 sealing only 12% of nicks, and K845N LIG1 displaying barely detectable levels of nick sealing (∼1%; Fig. 5*A*). Thus, K845N LIG1 maintains a similar level of enhanced discrimination against 8oxoG•A at 0.2 mM Mg^2+^ as was observed at 1 mM Mg^2+^. These observations indicate that LIG1 initiates AMP transfer to the 8oxoG•A nicked substrate but frequently dissociates from the AMP-DNA intermediate before completing nick sealing. The contribution of abortive ligation to ligation fidelity is discussed below.

## Discussion

As the primary ligase for DNA replication, recombination and repair, LIG1 must sustain high catalytic efficiency while preserving high fidelity to prevent genome instability. We demonstrated that reduced Mg^2+^ concentration within the physiological range results in decreased catalytic efficiency and elevated abortive ligation. This effect is seen with both WT LIG1 and the K845N variant that has been correlated with a delayed onset of HD. As different tissues are predicted to have a wide range of intracellular free Mg^2+^ concentration, we compared the catalytic activity of LIG1 variants at 0.2 mM Mg^2+^ with that previously reported at 1.0 mM Mg^2+^ (5). Here, we discuss the impact of K845N on the fidelity and catalytic efficiency of LIG1, its potential role in HD, and the broader implications for understanding the physiological impacts of rare LIG1 variants.

### LIG1 fidelity is enhanced at low Mg^2+^

The overall fidelity for discrimination against 8oxoG•A lesions, defined as the ratio of catalytic efficiencies for the C•G and 8oxoG•A substrates (Equation 5), is reported in Figure 5 and Table S3. At 1.0 mM Mg^2+^, WT LIG1 exhibits a discrimination factor of just 5.1 (Fig. 5*B*; Table S3). This reflects the difficulty that LIG1 has in discriminating against the 8oxoG•A Hoogsteen pairing which resembles the shape of Watson-Crick base pairs (15, 34). Similarly, DNA polymerases have difficulty in distinguishing 8oxoG•A pairs from Watson-Crick pairs (39, 40, 41). Reducing Mg^2+^ concentration to 0.2 mM enhances discrimination by WT LIG1 against the 8oxoG•A substrate by 21-fold (5.1- to 110-fold) (Fig. 5*B*; Table S3). K845N LIG1 exhibits a similar trend, with a 13-fold enhancement in fidelity at the lowered Mg^2+^ concentration (from 60- to 800-fold). These observations demonstrate that the overall fidelity of ligation is enhanced for both WT and K845N LIG1 under low Mg^2+^ conditions.

As overall fidelity is the product of fidelity of AMP transfer (step 2) and fidelity of nick sealing (step 3), we evaluated each individual step. Step 2 discrimination is calculated by including abortive ligation (*i.e.,* both abortive ligation and product formation) in the measurement of catalytic efficiency. At 1.0 mM Mg^2+^ step 2 discrimination is modest, at just 2.5 ± 0.7 for WT and 4.3 ± 1.5 for K845N LIG1 (Fig. 5*C*; Table S3). Lowering Mg^2+^ from 1.0 to 0.2 mM increases step 2 discrimination 5.6-fold for WT and 4.4-fold for K845N LIG1. These data indicate that the K845N LIG1 variant is only ∼2-fold more likely as WT LIG1 to reject 8oxoG•A substrates at the AMP transfer step regardless of Mg^2+^ concentration. Therefore, the impact of the K845N substitution is largely independent of contribution from reduced concentration of Mg^2+^.

Step 3 discrimination describes fidelity at the nick sealing step and is calculated through Equation 7. At 1.0 mM Mg^2+^, step 3 discrimination is low for WT LIG1 (2.0 ± 0.1), indicating that once AMP transfer occurs, 8oxoG•A nicks are frequently sealed (Fig. 5*D*; Table S3). For K845N LIG1 step 3 discrimination is substantially higher (13.9 ± 1.7), demonstrating enhanced rejection of 8oxoG•A substrates following AMP transfer. This difference suggests that while K845N LIG1 has higher fidelity than WT LIG1 at both step 2 and step 3, overall discrimination exhibited by the K845N LIG1 variant is mostly driven by a fidelity enhancement at the nick sealing step of the LIG1 mechanism. Reducing the Mg^2+^ concentration from 1.0 to 0.2 mM enhances step 3 discrimination by similar levels for both WT (4.0-fold) and K845N (3.1-fold), again demonstrating that the effect due to reduced Mg^2+^ is largely independent of the increased fidelity of K845N LIG1.

**Figure 6:**
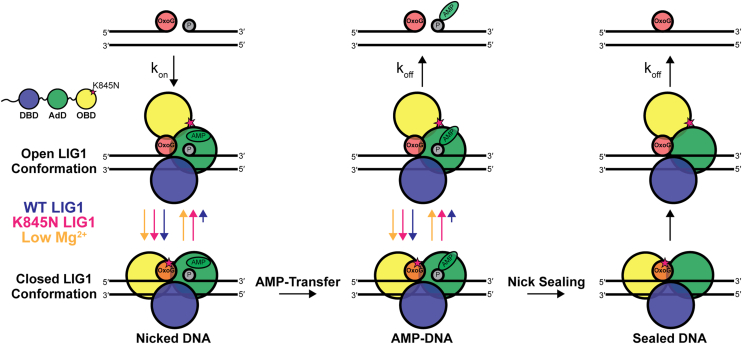
**Model for how the K845N variant and/or limiting Mg^2+^ modulates DNA ligation fidelity.** We propose that an open to closed binding transition of LIG1 contributes to discrimination against the 3′ mismatch (enhanced fidelity). In the closed conformation the OBD (*yellow domain*) enables LIG1 to fully encircle the DNA substrate. Relative rates for opening and closing are indicated by *colored arrow* lengths. The enhanced discrimination at step 2 (AMP-transfer) and step 3 (nick sealing) can be explained by an increased opening of the OBD and subsequent dissociation. This shift is due to slowing of the chemical steps and/or increased dynamics of the closed complex. LIG1 dissociation is enhanced on the mismatch before the transfer of AMP onto the 5′- phosphate group of the nicked substrate *(left)*. When adenylylation of the nicked substrate occurs, K845N and low Mg^2+^ promote increased dissociation of LIG1 from the AMP-DNA intermediate, leading to abortive ligation events *(middle)*. If LIG1 remains in the closed conformation after AMP transfer, the nick sealing reaction proceeds normally, and LIG1 and sealed DNA dissociate *(right)*. LIG1, DNA ligase I.

A model for how fidelity of ligation is enhanced by limiting concentration of Mg^2+^ ions is shown in Figure 6. We propose that the relative equilibrium for opening of the LIG1 complex is enhanced at limiting Mg^2+^ due both to destabilization of the closed complex and by slowing of the chemical steps (yellow arrows; Fig. 6). This provides greater opportunity for rejection of the 8oxoG•A mismatch in both the initial complex of LIG1 with the nicked DNA substrate (Fig. 6; left) and in the complex with the AMP-DNA intermediate after AMP-transfer (Fig. 6; middle). Further work is needed to understand the extent to which structural dynamics of the OBD may differ between matched and mismatched DNA substrates, however there are clues that steric strain imposed by the HiFi Mg^2+^ site is important for the positioning of the nick in the active site (15) and a range of dynamics have been observed for LIG1 using molecular dynamics in the case of a canonical DNA nick (12).

The fidelity enhancement conferred by the K845N substitution is also shown in Figure 6. We assume that the initial association of LIG1 with nicked DNA is comparable for WT and K845N LIG1 as K845 will not be engaged with interdomain contacts in an open complex. The WT enzyme stably closes around the nicked site, aided by favorable interactions between K845 and backbone carbonyls of L750 and V753 observed in crystal structures of LIG1 bound to DNA (Fig. S1; (15)). For K845N LIG1, it is likely that the equilibrium for the closed complex shifts toward a more open complex to increase the frequency of dissociation. Following AMP transfer, a similar closed *versus* open equilibrium can exist and again the K845N variant is expected to spend more time in the open complex (Fig. 6; middle). Subsequent release from the open complex leads to abortive ligation and rapid adenylylation of LIG1 by ATP. The closed complex can subsequently perform nick sealing to form the ligated DNA product.

In the case of abortive ligation, DNA ligases cannot immediately re-engage because the high concentration of cellular ATP drives reformation of the adenylylated enzyme intermediate. Thus, fidelity at the nick sealing step has the potential to cause persistent breaks that could be deleterious, particularly in replicating cells. However, there are multiple pathways to process the AMP-DNA species. The most direct is hydrolysis by aprataxin (APTX) which regenerates the ligatable nick. A second pathway is strand displacement synthesis followed by flap endonuclease cleavage to generate a new ligatable nick. Either pathway would cause a delay allowing for further processing of the DNA immediately upstream or downstream of the break and ultimately another attempt at ligation which could increase the overall fidelity of repair (16, 42, 43). Deficiency in APTX causes a specific neurodegenerative disease, with dysfunction and death primarily of cerebellar Purkinje cells. It has been suggested that this disease is due to decreased ability of these cells to handle AMP-DNA intermediates, particularly in the context of oxidative damage (42). Although Purkinje cells are lost to some extent in Huntington’s disease (44), the primary degeneration occurs in the medium-spiny neurons of the striatum and APTX is not a known modifier of HD. Furthermore, little is known about frequency of DNA damage and repair pathway choice in differentiated neurons.

The observation that abortive ligation is enhanced and nick sealing is impaired at low Mg^2+^ is consistent with previous work, which established that Mg^2+^ affinity is high during step 2, and lowest during step 3 (16). Therefore, AMP transfer can still occur at low Mg^2+^, albeit with greatly impaired nick sealing, resulting in increased abortive ligation. Our data confirms this observation by showing a ∼3-fold decrease in k_cat_ and k_cat_/K_M_ for WT LIG1 at 0.2 mM *versus* 1.0 mM Mg^2+^, while K845N LIG1 experiences a more severe catalytic impairment at low Mg^2+^ (5-fold decrease in k_cat_, 11-fold in k_cat_/K_M_) for a canonical nicked DNA substrate (Fig. 4, *A* and *C*; Table S3). Nevertheless, WT LIG1, and to a lesser degree K845N LIG1, retain significant activity at the lowest estimated physiological Mg^2+^ concentration, suggesting that these LIG1 variants are efficient DNA ligases under most physiological contexts.

### Implications for the protective role of K845N in HD

The K845N LIG1 variant is correlated with a delayed onset of symptomatic HD in heterozygous patients, suggesting a protective role in the context of this neurodegenerative disorder that is characterized by repeat expansion due to errors in DNA repair (4, 5, 45, 46). In later stages of HD, endoplasmic reticulum dysfunction increases oxidative stress which also elevates the need for DNA repair (47). Recent kinetic characterization of K845N at a free Mg^2+^ concentration of 1.0 mM demonstrated that this variant is a higher fidelity enzyme, better able to reject mismatched and damaged DNA nicks as compared to WT LIG1 (5). In a mouse model for K843N (analogous to K845N of human LIG1), there is a statistically significant decrease in the extent of CAG repeat expansion (5). In the current work we extended these earlier findings to dissect the magnesium dependence of DNA ligation at reduced concentration of free Mg^2+^ ions that may be as low as 0.2 mM in the brain. We found that the fidelity of WT LIG1 is increased under conditions of reduced Mg^2+^, and that the K845N variant retains the higher fidelity that was observed at higher concentration of Mg^2+^. We hypothesize that K845N LIG1 is protective against mutagenesis and/or repeat expansions in disease-relevant tissues, however further experimentation is required to test these hypotheses *in vivo*. As there are many potential DNA structures relevant to expansion or contraction of CAG repeats, it will be important to consider whether the higher fidelity of K845N in the context of oxidative damage might extend to some of these expanded CAG repeat structures.

The biochemical characterization of K845N LIG1 suggests a mechanism by which it could afford protection in the heterozygous context. If K845N primarily enhanced discrimination at step 2, then it is unlikely that K845N could outcompete a WT version of LIG1. However, K845N enhances discrimination primarily at step 3 with the release of the AMP-DNA intermediate. This intermediate is not available for ligation by WT LIG1 because LIG1 binds tightly and reacts quickly with ATP to regenerate the AMP-LIG1 intermediate. Cells have multiple pathways to remove blocked 5′-ends, but regardless of whether this occurs *via* polymerase strand displacement and flap endonuclease cleavage or *via* direct removal of the 5′-AMP group by APTX, it is expected that the extended lifetime of the nicked DNA species will provide extended opportunities for exonucleolytic removal of damaged, mismatched or slipped intermediates at the 3′-hydroxyl side of the nick. These considerations suggest that the homozygous inheritance of K845N would further enhance the delay of onset of HD.

### Implications for LIG1 syndrome

LIG1 syndrome is a very rare primary immune deficiency characterized by biallelic hypomorphic variants of LIG1. The clinical symptoms vary widely in severity amongst individuals, and it is not yet clear if these differences are due to the specific alleles involved or to involvement of other genetic factors. In LIG1 syndrome patients there is a profound reduction in gamma globulins and in multiple types of lymphocytes (2). It is notable that T-cells resemble neuronal cells in having very low levels of intracellular free Mg^2+^ ions (48, 49), which makes Mg^2+^ availability an important consideration when evaluating LIG1 function in these tissues.

Despite occurring in approximately 1 in 700 individuals (gnomAD v.4.1.0), the K845N LIG1 variant does not appear to be correlated with LIG1 syndrome, likely because its reduced catalytic activity is still sufficient to support normal immune cell function. Our data demonstrate that while LIG1 fidelity is greatly enhanced at low Mg^2+^ levels, LIG1 catalytic activity is reduced, which carries important implications for understanding LIG1 syndrome. LIG1 syndrome variants typically exhibit reduced Mg^2+^ utilization and increased K_1/2_ for Mg^2+^, making them especially vulnerable to a low Mg^2+^ environment (30, 31). As both immune and neuronal cells function under lower Mg^2+^ concentrations, it is essential to evaluate LIG1 variants associated with LIG1 syndrome and HD within this context of limiting Mg^2+^ to better understand the impact of these rare amino acid substitutions in the disease-relevant tissues. Without such characterization there is the risk that important functional nuances may be overlooked as they are suppressed at higher concentration of Mg^2+^.

## Experimental procedures

### Preparation of oligonucleotides

Oligonucleotides used in experiments were obtained from IDT and purified *via* denaturing PAGE. Absorbance values were obtained *via* UV spectroscopy, and concentrations were calculated *via* Beer’s law with extinction coefficients at 260 nm. Annealed DNA substrates were prepared by combining oligonucleotides with a 5′ phosphate group with a 3′ FAM label, 3′ OH strand, and a complementary template strand in a 1:1.5:2 ratio, corresponding to concentrations of 10 μM phosphate strand (FAM-labeled), 15 μM template, and 20 μM OH strand. Oligos were mixed in annealing buffer (10 mM MES buffer pH 6.5 and 50 mM NaCl). Once combined, solutions were heated to 95 °C, and then slowly cooled to 4 °C at a rate of 1 °C every 5 s. Nicked DNA substrates were stored at 4 °C.

### Preparation of LIG1 protein

Δ232 LIG1 variants were prepared as previously described (5). Briefly, WT and K845N LIG1 variants were cloned into a pET19 vector, and overexpressed in *E. coli* using auto-induction in TB media. Cells were lysed and Δ232 LIG1 was purified using Ni-NTA affinity chromatography followed by anion exchange chromatography (HiTrap Q). LIG1 was adenylylated and the N-terminal His-Tag was cleaved with PreScission protease. Purified proteins were stored at −80 °C in enzyme storage buffer (25 mM Tris-Cl pH 7.5, 150 mM NaCl, 0.1 mM EDTA, 1 mM DTT). Protein purity was verified by SDS-PAGE.

### Gel-based ligation assays

The active concentration of LIG1 was determined using the active site titration assay. A range of 25 nM to 150 nM Δ232 LIG1 variants were incubated with 100 nM nicked DNA substrate in the absence of ATP to prevent enzyme turnover. Reactions took place in active site titration reaction buffer (50 mM MOPS pH 7.5, 1 mM DTT, 0.5 mg/ml BSA, 10 mM MgCl_2_, ionic strength adjusted to 150 mM using NaCl) at 37 °C for 1 h. Reactions were quenched with a standard loading buffer (90% formamide, 50 mM EDTA, 0.01% bromophenol blue, and 0.01% xylene cyanol) followed by heating at 95 °C for 5 min. Ligated DNAs were separated from nicked substrate *via* denaturing PAGE (15% polyacrylamide/8 M urea). Fluorescein labeled nicked substrate and sealed product were detected using the Amersham Typhoon 5 imager and band intensities were quantified with ImageQuant TL software (Cytiva; www.cytivalifesciences.com). Active enzyme concentration was calculated using segmental linear regression analysis in GraphPad Prism 10 software (www.graphpad.com).

Steady-state kinetics experiments were performed at 37 °C in a standard reaction buffer (50 mM MOPS pH 7.5, 1 mM DTT, 0.5 mg/ml BSA, 0.2 mM ATP, varying MgCl_2_ concentrations and ionic strength adjusted to 150 mM using NaCl) and varying concentrations of LIG1 and DNA. To determine the nicked substrate dependence on ligase activity, steady-state assays were performed using an excess of C•G or 8oxoG•A 28mer nicked DNA substrate (5–1000 nM) and limiting enzyme (0.01–16 nM for WT and 0.02–32 nM for K845N LIG1) such that multiple-turnover velocities are determined by at least 5 enzyme turnovers. At specified time points, reactions were quenched and quantified as above. Initial rates were determined by fitting the fraction product (Equation 1) with linear fits in GraphPad Prism, from which reaction velocities were calculated (*p* is ligated product, *s* is unreacted nicked DNA substrate, and *i* is AMP-DNA intermediate). The fraction of abortive ligation was determined (Equation 2), and initial rates (<0.20) of sealed product formation were fit by the Michaelis-Menten equation to determine k_cat_ and K_M_ values (Equation 3), as well as the catalytic efficiency (k_cat_/K_M_). For assays determining LIG1 dependence on free Mg^2+^, k_cat_, $K_{\text{Mg}}$ and k_cat_/ $K_{\text{Mg}}$ Equation 4 was used. The overall discrimination between 8oxoG•A-containing substrate and undamaged (C•G) substrate was calculated using Equation 5 in which the catalytic efficiency was calculated for formation of ligated product. Fraction sealed product was determined using Equation 6. The discrimination in step 3 is calculated from the fraction sealed for canonical and mismatched substrates using Equation 7. The overall discrimination is the product of discrimination in step 2 and step 3 (Equation 8), which can be used to calculate the discrimination in step 2. Error in the discrimination measurements was determined using the sum of squares error propagation.

(1) $$F_{p}=\frac{p}{p+s+i}$$

(2) $$F_{abort}=\frac{i}{p+i}$$

(3) $$\frac{V_{init}}{\left[E\right]}=\frac{V_{\max}\left[S\right]}{K_{M}+\left[S\right]}$$

(4) $$\frac{V_{init}}{\left[E\right]}=\frac{V_{\max}\left[{Mg}^{2+}\right]}{K_{Mg}+\left[{Mg}^{2+}\right]}$$

(5) $$Discrimination_{Overall}=\frac{{Catalytic\;Efficiency}_{C\cdot G}}{{Catalytic\;Efficiency}_{8oxoG\cdot A}}$$

(6) $$F_{sealed}={1-F}_{Abort}=\frac{p}{p+i}$$

(7) $${Discrimination}_{Step3}=\left(F_{sealed\;C\cdot G}\right)\;/\;\left(F_{sealed\;8oxoG\cdot A}\right)$$

(8) $${Discrimination}_{Overall}=\left({Discrimination}_{Step2}\right)*\left({Discrimination}_{Step3}\right)$$

## Data availability

All data can be found within the manuscript and accompanying supporting information.

## Supporting information

This article contains supporting information (1).

## Conflict of interest

The authors declare that they have no conflicts of interest with the contents of this article.
