# Supplementary material for: Fidelity of DNA ligase I is sensitive to physiological Mg2+ level
Source: J Biol Chem. 2026 Mar 25;302(5):111407. doi: 10.1016/j.jbc.2026.111407 (PMC13126024; doi:10.1016/j.jbc.2026.111407)
Supplement: Supporting Figures and Tables [file mmc1.pdf]

Supporting Information

Fidelity of DNA Ligase I is sensitive to physiological Mg^{2+} level

David H. Beier, Eunhye Lee, Ihn Sik Seong, Vanessa C. Wheeler, and Patrick J. O'Brien*

*Correspondence: pjobrien@umich.edu

Table of Contents

Table S1. Oligonucleotides for ligation assays p.S2
Table S2. Steady-State Kinetic Parameters and Fidelity Measurements for Δ232 LIG1 p.S3
Table S3. Kinetic parameter comparison between Mg^{2+} concentrations p.S4
Figure S1. K845 in the OBD makes hydrogen bonding contacts with the AdD p.S5
Figure S2. Steady-state free magnesium dependence of WT and K845N LIG1 p.S6
Figure S3. Alternative salt conditions do not alter Mg^{2+} dependence of LIG1 p.S7
Figure S4. Enzyme turnovers for sealed and abortive product on canonical substrate p.S8
Figure S5. Enzyme turnovers for sealed and abortive product on oxidized substrate p.S9

Oligo Name	5' Mod	Sequence	3' Mod
Up13OH-C		5' GTGCTGATGCGTC 3'	
UP13OH-oxoG		5' GTGCTGATGCGT^{oxo}G 3'	
DownP15-FAM	Phosphate	5' P-GTCGGACTGATTCGG-FAM 3'	Fluorescein (6-FAM)
Temp28-G		5' CCGAATCAGTCCGACGACGCATCAGCAC 3'	
Temp28-A		5' CCGAATCAGTCCGAC<u>A</u>ACGCATCAGCAC 3'	

Table S1: Oligonucleotides for ligation assays. Up13OH-C, DownP15-FAM and Temp28-G were annealed to generate the canonical 28mer DNA substrate. Up13OH-oxoG, DownP15-FAM and Temp28-A were annealed to generate the 8oxoG•A mismatch DNA substrate. Oligos were annealed in a 1:1.5:2 ratio (DownP15-FAM: Temp28: Up13OH).

C•G Nicked DNA

	WT	K845N	*WT/K845N*
k_{cat} (s^{-1})	0.21 ± 0.04	0.04 ± 0.01	*5.3*
K_M (nM)	55 ± 16	168 ± 27	*0.33*
k_{cat}/K_M ($M^{-1}s^{-1}$)	3.9 ± 0.7 x10^6	0.22 ± 0.02 x10^6	*18*
Fraction Sealed	0.96 ± 0.02	0.55 ± 0.08	

8oxoG•A Nicked DNA

	WT	K845N	*WT/K845N*
k_{cat} (s^{-1})	5.0 ± 0.2 x10^{-3}	12 ± 0.6 x10^{-5}	*42*
K_M (nM)	142 ± 27	426 ± 37	*0.33*
k_{cat}/K_M ($M^{-1}s^{-1}$)	0.04 ± 0.01 x10^6	0.2 ± 0.04 x10^3	*133*
Fraction Sealed	0.12 ± 0.01	0.013 ± 0.004	

Fidelity Measurements

	WT	K845N	*K845N Enhancement*
Overall Discrimination	110 ± 30	800 ± 100	*7.3*
Discrimination in Step 2	13 ± 5	18 ± 7	*1.4*
Discrimination in Step 3	8 ± 2	43 ± 17	*5.4*

Table S2: Steady-State Kinetic Parameters and Fidelity Measurements for Δ232 LIG1. These data were collected from experiments performed in standard reaction buffer with 0.2 mM free Mg^{2+}. Values are the average ± standard deviation with a minimum of 3 replicates.

C•G Nicked DNA

		k_{cat} (s^{-1})	K_M (nM)	k_{cat}/K_M ($M^{-1}s^{-1}$)	Fraction Sealed
**WT**	1 mM Mg^{2+}	0.52 ± 0.01	42 ± 6	13 ± 2 x10^6	0.99 ± 0.01
	Rel. to 0.2 mM	2.5	0.76	3.3	1.0
**K845N**	1 mM Mg^{2+}	0.20 ± 0.01	81 ± 14	2.5 ± 0.4 x10^6	0.97 ± 0.01
	Rel. to 0.2 mM	5	0.48	11.4	1.8

8oxoG•A Nicked DNA

		k_{cat} (s^{-1})	K_M (nM)	k_{cat}/K_M ($M^{-1}s^{-1}$)	Fraction Sealed
**WT**	1 mM Mg^{2+}	0.08 ± 0.01	31.7 ± 5.9	2.4 ± 0.5 x10^6	0.49 ± 0.03
	Rel. to 0.2 mM	16	0.2	60	4.1
**K845N**	1 mM Mg^{2+}	3.0 ± 0.5 x10^{-3}	73 ± 17	0.04 ± 0.01 x10^6	0.07 ± 0.01
	Rel. to 0.2 mM	27	0.2	150	5.4

Fidelity Measurements

	**WT**	**K845N**
**Overall Discrimination**		
1 mM Mg^{2+}	5.1 ± 1.3	60 ± 20
Enhancement at 0.2 mM	21	13
**Discrimination in Step 2**		
1 mM Mg^{2+}	2.5 ± 0.7	4.3 ± 1.5
Enhancement at 0.2 mM	5.6	4.4
**Discrimination in Step 3**		
1 mM Mg^{2+}	2.0 ± 0.1	13.9 ± 1.7
Enhancement at 0.2 mM	4.0	3.0

Table S3: Kinetic parameter comparison between Mg^{2+} concentrations. The previously determined (1) kinetic parameters for the standard reaction buffer with 1.0 mM free Mg^{2+} are tabulated. Relative values are the ratios of these values and the values for 0.2 mM free Mg^{2+} from Table S2.

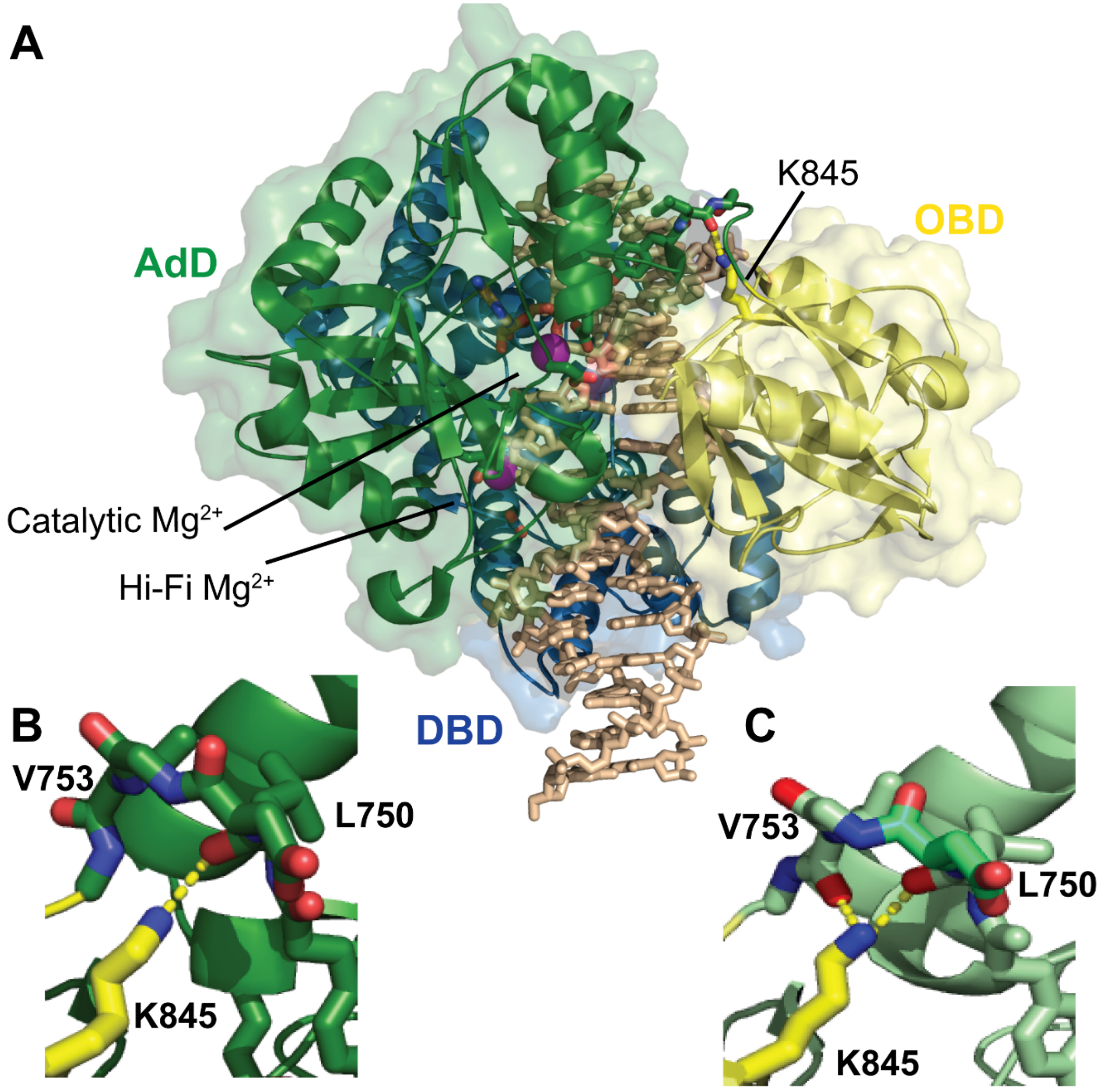

Figure S1. K845 in the OBD makes hydrogen bonding contacts with the AdD. *A*, LIG1 in complex with the dideoxy terminated AMP-DNA intermediate in the presence of 200 mM $MgCl_2$ (PDB: 6P09). Mg^{2+} ions in the active site and HiFi site are shown as purple spheres. *B*, Hydrogen bond between the epsilon amino of K845 and the backbone carbonyl of L750 from 6P09. *C*, Hydrogen bonds between the epsilon amino of K845 and the backbone carbonyls of L750 and V753 from PDB:6P0C, a structure of LIG1 in complex with AMP-DNA in the presence of EDTA. A bifurcated hydrogen bond is seen in the majority of LIG1 structures and may be disrupted in the 6P09 structure due to the very high Mg^{2+} concentration, as a similar structure at 2 mM Mg^{2+} (PDB: 6P0A) also shows the bifurcated hydrogen bonding (not shown). Images were rendered in Pymol (Schrodinger, LLC) and structures were determined by Tumbale et. al (2).

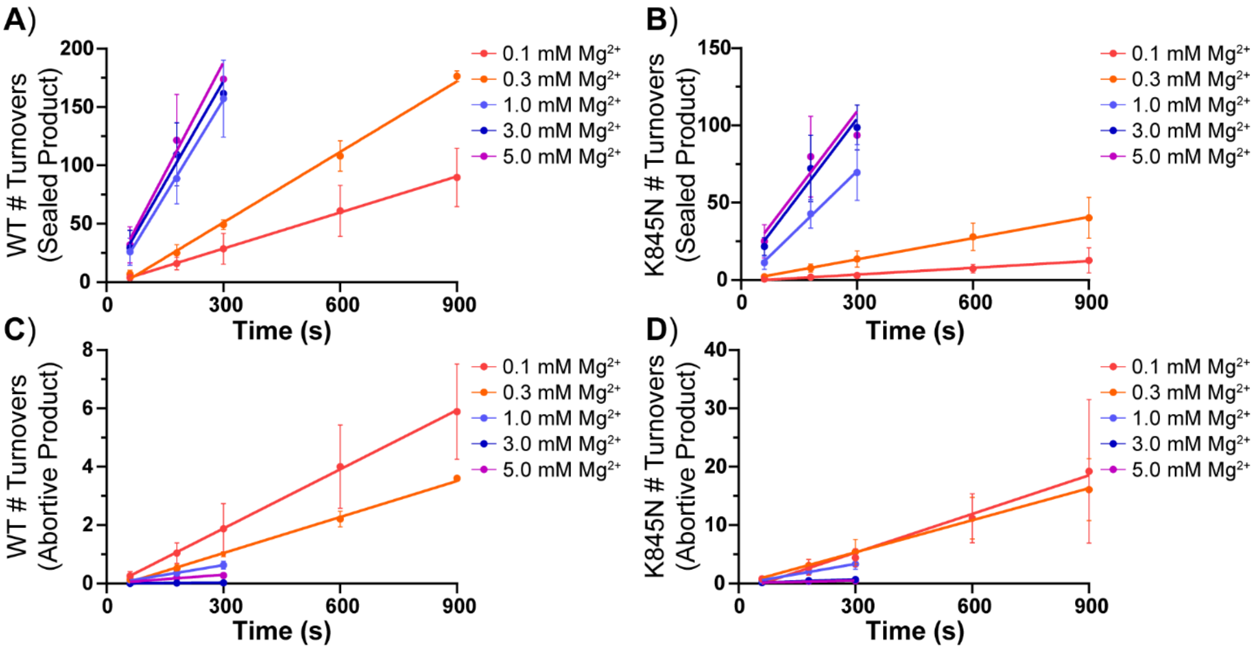

Figure S2. Steady-state free magnesium dependence of WT and K845N LIG1. Initial rates for sealed product formation were determined using 1 - 2 nM LIG1, 0.2 mM ATP, 1000 nM nicked DNA substrate and 0.1 - 5.0 mM free [Mg^{2+}] for WT *(A)* and K845N *(B)*. These initial rates were fit by **Equation 5** to determine k_{cat} and $K_{Mg^{2+}}$ values. Initial rates for abortive ligation were also determined for WT *(C)* and K845N *(D)*. Data reported are the average and standard deviation of at least 3 replicates.

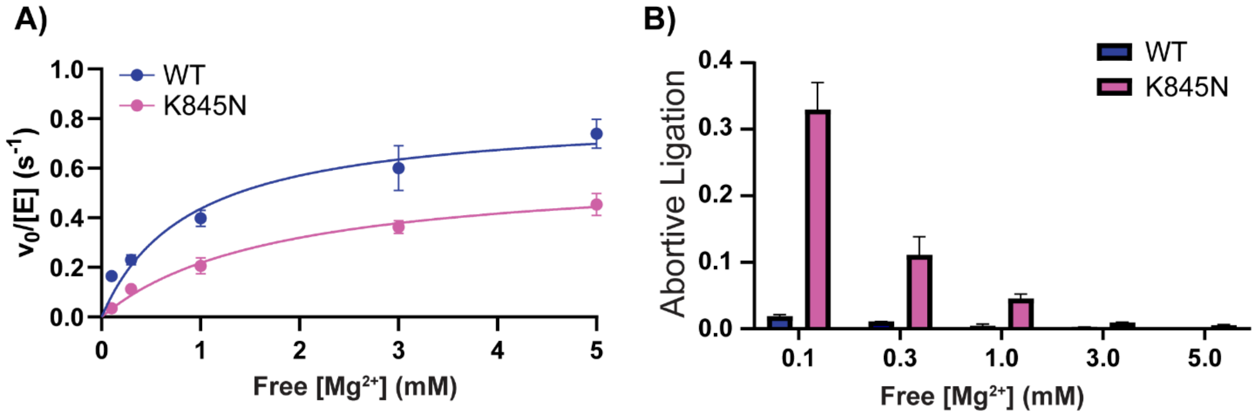

MgOAc₂ Dependence

	k_{cat} (s^{-1})	K_{Mg} (mM)	k_{cat}/K_{Mg} ($M^{-1}s^{-1}$)
**WT**	0.82 ± 0.13	0.85 ± 0.25	960 ± 320 x10^6
**K845N**	0.60 ± 0.06	1.8 ± 0.2	340 ± 52 x10^6

Figure S3. Alternative salt conditions do not alter Mg^{2+} dependence of LIG1. Reactions were performed at 37 °C with 0.2 mM ATP, variable $MgOAc_2$, 50 mM MOPS pH 7.5 and an ionic strength of 150 mM adjusted with NaOAc. *A,* Steady-state dependence on Mg^{2+} concentration was fit by a rectangular hyperbola to determine the best fit values of the apparent binding constant for a single Mg^{2+} ion as $K_{Mg^{2+}}$. k_{cat} values for WT and K845N were determined to be 0.82 ± 0.13 and 0.60 ± 0.06 s^{-1}, respectively. *B,* The fraction abortive ligation was calculated at each free Mg^{2+} concentration. Data reported are the average and standard deviation of 3 replicates.

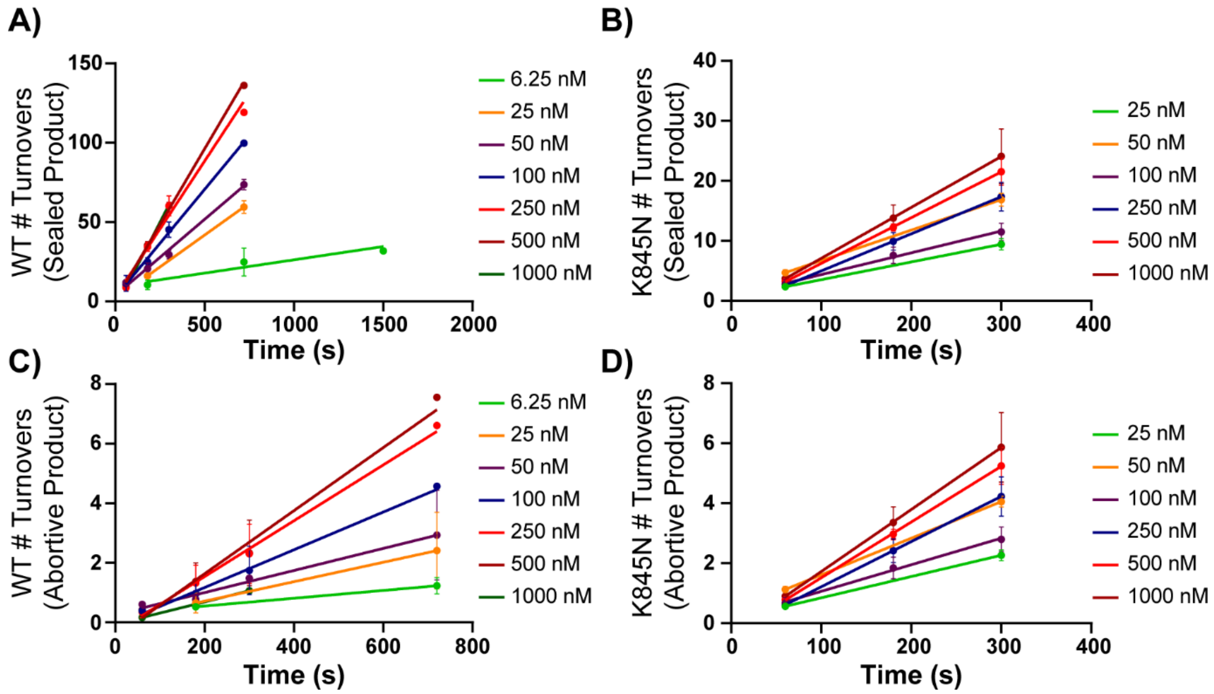

Figure S4. Enzyme turnovers for sealed and abortive product on canonical substrate. Initial rates were determined using 0.33 – 3.33 nM Δ232 WT LIG1 or 0.33 - 5 nM Δ232 K845N LIG1, 0.2 mM ATP, 6.25 - 1000 nM nicked DNA substrate and 0.2 mM free [Mg^{2+}]. Initial rates for sealed product formation of the canonical 28mer DNA substrate were converted to turnover number as [Sealed Product] / [Enzyme] to account for the different enzyme concentrations for WT *(A)* and K845N *(B)*. Turnover number for release of intermediate as abortive product was calculated as [Abortive Product] / [Enzyme] for WT *(C)* and K845N *(D)*. Data reported are the average and standard deviation of at least 3 replicates.

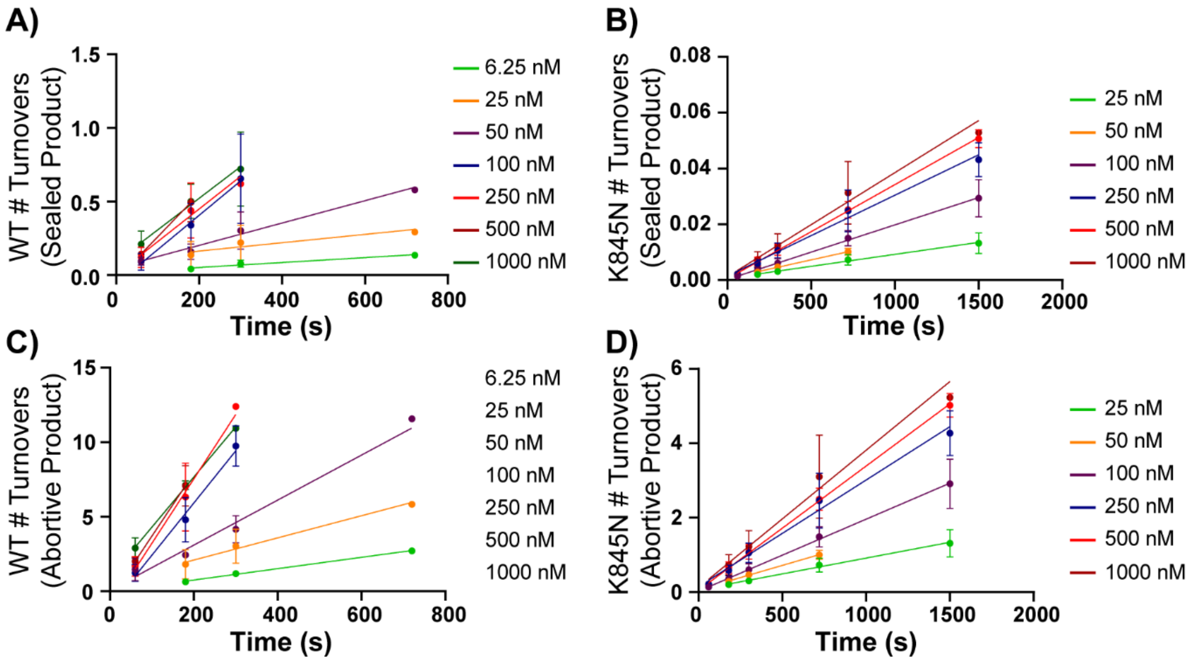

Figure S5. Enzyme turnovers for sealed and abortive product on 8oxoG•A substrate. Initial rates were determined using 0.5 - 30 nM Δ232 WT LIG1 or 0.5 - 30 nM Δ232 K845N LIG1, 0.2 mM ATP, 6.25 - 1000 nM nicked DNA substrate and 0.2 mM free [Mg^{2+}]. Initial rates for sealed product formation of the 8oxoG•A mismatch substrate were converted to turnover number as [Sealed Product] / [Enzyme] to account for the different enzyme concentrations for WT *(A)* and K845N *(B)*. Turnover number for release of intermediate as abortive product was calculated as [Abortive Product] / [Enzyme] for WT *(C)* and K845N *(D)*. Data reported are the average and standard deviation of at least 3 replicates.

Supporting References

1. Lee, E., Kim, W., Beier, D. H., Lee, Y., Kovalenko, M., Saif, F. *et al.* (2026) Huntington's disease LIG1 modifier variant increases ligase fidelity and suppresses somatic CAG repeat expansion *Proc Natl Acad Sci U S A* **123**, e2518854123
2. Tumbale, P. P., Jurkiw, T. J., Schellenberg, M. J., Riccio, A. A., O'Brien, P. J., and Williams, R. S. (2019) Two-tiered enforcement of high-fidelity DNA ligation *Nat Commun* **10**, 5431
